# Besifloxacin liposomes with positively charged additives for an improved topical ocular delivery

**DOI:** 10.1038/s41598-020-76381-y

**Published:** 2020-11-06

**Authors:** Giselly Almeida dos Santos, Ricardo Ferreira-Nunes, Luciana Facco Dalmolin, Ana Carolina dos Santos Ré, Jorge Luiz Vieira Anjos, Sebastião Antônio Mendanha, Carolina Patrícia Aires, Renata F. V. Lopez, Marcilio Cunha-Filho, Guilherme M. Gelfuso, Taís Gratieri

**Affiliations:** 1 Laboratory of Food, Drugs, and Cosmetics (LTMAC), University of Brasilia, Brasília, DF 70910-900 Brazil; 2 School of Pharmaceutical Sciences of Ribeirão Preto, University of São Paulo, Ribeirão Preto, SP 14040-903 Brazil; 3 Institute of Physics, Federal University of Goiás, Catalão, GO 75704-020 Brazil; 4 Institute of Physics, Federal University of Goiás, Goiânia, GO 74690-900 Brazil

**Keywords:** Drug delivery, Eye diseases, Corneal diseases

## Abstract

Topical ophthalmic antibiotics show low efficacy due to the well-known physiological defense mechanisms of the eye, which prevents the penetration of exogenous substances. Here, we aimed to incorporate besifloxacin into liposomes containing amines as positively charged additives and to evaluate the influence of this charge on drug delivery in two situations: (i) iontophoretic and (ii) passive treatments. Hypothesis are (i) charge might enhance the electromigration component upon current application improving penetration efficiency for a burst drug delivery, and (ii) positive charge might prolong formulation residence time, hence drug penetration. Liposomes elaborated with phosphatidylcholine (LP PC) or phosphatidylcholine and spermine (LP PC: SPM) were stable under storage at 6 ºC for 30 days, showed mucoadhesive characteristics, and were non-irritant, according to HET-CAM tests. Electron paramagnetic resonance spectroscopy measurements showed that neither the drug nor spermine incorporations produced evident alterations in the fluidity of the liposome's membranes, which retained their structural stability even under iontophoretic conditions. Mean diameter and zeta potential were 177.2 ± 2.7 nm and − 5.7 ± 0.3 mV, respectively, for LP PC; and 175.4 ± 1.9 nm and + 19.5 ± 1.0 mV, respectively, for LP PC:SPM. The minimal inhibitory concentration (MIC) and the minimal bactericide concentration (MBC) of the liposomes for *P. aeruginosa* showed values lower than the commercial formulation (Besivance). Nevertheless, both formulations presented a similar increase in permeability upon the electric current application. Hence, liposome charge incorporation did not prove to be additionally advantageous for iontophoretic therapy. Passive drug penetration was evaluated through a novel in vitro ocular model that simulates the lacrimal flow and challenges the formulation resistance in the passive delivery situation. As expected, LP PC: SPM showed higher permeation than the control (Besivance). In conclusion, besifloxacin incorporation into positively charged liposomes improved passive topical delivery and can be a good strategy to improve topical ophthalmic treatments.

## Introduction

Bacterial conjunctivitis and keratitis are inflammation of conjunctiva and cornea tissues, respectively, caused by a bacterial infection^1–3^. Common signs are redness of the eye and purulent secretion^1,2^. Fluoroquinolones are the class of antibiotics mainly used for treating these conditions because of their broad-spectrum activity, including gram-positive, gram-negative, and anaerobic organisms, as well as their documented safety and low toxicity^4^.

The Food and Drug Administration (FDA) approved formulation for the treatment of bacterial conjunctivitis is a besifloxacin topical suspension at 0.6% (Besivance; Bausch + Lomb, Rochester, NY). This medicine is composed of a polymeric mucoadhesive system for release control (DuraSite; InSite Vision, Inc., Alameda, CA), which might prolong the drug’s contact with the ocular surface and increase its concentration in the conjunctiva^5,6^. Indeed, the major problem with topical ophthalmic therapies, however, is the poor drug bioavailability due to the own ocular anatomy and physiology, which fast remove part of the formulation from the ocular surface, reducing drug residence time and, consequently, absorption^7^. However, this system (DuraSite), composed of benzalkonium chloride 0.01%, polycarbophil, mannitol, poloxamer 407, sodium chloride, and disodium dihydrate edetate presents a high viscosity being uncomfortable to instill and bringing other inconvenient as blurred vision and dose variability^8–10^.

An option for increasing drug bioavailability with no pain or discomfort could be the use of physical enhancement technologies applied in the clinical environment by a trained professional. We have recently suggested using iontophoresis as “an emergency burst delivery approach” for the treatment of ocular fungal infections^11^. The main argument was that iontophoresis might help to achieve therapeutic concentrations more quickly—thereby providing faster treatment and symptom relief. The rationale is to apply the iontophoresis in the clinic immediately after the diagnose, so a significant antibiotic dose is promptly achieved at the infection site, and then the patient can continue with the conventional application of a drug delivery system at home, until necessary. With such technique, drug permeation is enhanced by the sum of three-component mechanisms: passive diffusion, electromigration and electroosmosis^12–15^, in which electromigration is the ordered movement of charged molecules in the presence of current and electroosmosis can be described as the solvent flow driven by an electric potential applied across an ionized membrane. Nevertheless, for small molecules with a significant passive component, the role of electroosmosis is less meaningful^16^. Thus, iontophoresis is a most suitable technology when the drug is charged at physiological pH, so the electromigration takes place. Nonetheless, when the drug net charge is zero at physiological pH, as is the case of besifloxacin (pKa strongest acidic 5.4 and pKa strongest basic 9.67), imparting charge to nanocarriers as means of improving electromigration, in theory, is a promising strategy. Such an approach has been evaluated in some cutaneous experiments, but, to date, results obtained are ambiguous^17–20^. To our knowledge this is the first time such strategy is applied for ocular drug delivery.

Moreover, the use of lipid vesicle systems, such as liposomes, can facilitate the transport across the cornea, increasing penetration and deposition into the ocular region, without the discomfort caused by viscous gels^7,9^. Accordingly, many liposomes have been proposed for ocular drug delivery^11,21^. Concerning the passive drug delivery, a commonly used strategy is the inclusion of positively charged polymers in the formulation or coating the nanosystem so that a mucoadhesive characteristic is conferred, which could increase formulation resistance to tear flow and drainage, ultimately increasing drug penetration and bioavailability^22–25^. Nevertheless, the drawback is such polymers might add viscosity to the formulation, bringing all those aforementioned inconvenient.

The addition of small positive molecules could be an alternative for imparting liposomes positive charge while maintaining hydrodynamic size and formulation viscosity^18,26,27^. But again, to date, such strategy has not been applied for ocular drug delivery.

Therefore, the first goal of this paper is to incorporate besifloxacin into liposomes containing amines as positive charge additives and to evaluate the influence of charge in the ocular iontophoresis, which could be used as an emergency “burst drug delivery” approach. Liposomes without the charge additives were also obtained for comparison purposes. The second goal of this paper was to evaluate the influence of the positive charge on the passive drug delivery. To demonstrate a possible system superiority, the drug delivery experiment must be capable of challenging the formulation resistance somehow, mimicking the physiological ocular defense mechanisms. For this, a novel in vitro ocular model that simulates the lacrimal flow was used to evaluate the passive application.

## Material and methods

### Material

Besifloxacin (> 99%, MW 430.301 g/mol, Log P 0.54, Water solubility: 0.143 mg/mL), spermine (SPM) (MW 202.34 g/mol), stearylamine (SA) (MW 269.51 g/mol), chloroform, mucin from porcine stomach (type III), spin label 5-doxyl-stearic acid (5-DSA), (2,2,6,6-tetramethylpiperidin-1-yl) oxidanyl (TEMPO) and resazurin sodium salt were purchased from Sigma-Aldrich (St. Louis, MO, USA). Cholesterol (CH) and soybean phosphatidylcholine (PC) was purchased from Avanti Polar Lipids (Alabaster, AL, USA). Buffer 4-(2-hydroxyethyl)-1piperazineethanesulfonic acid (HEPES) was purchased from Dinâmica (São Paulo, Brazil). Sodium chloride was purchased from Cromoline (São Paulo, SP, Brazil). Dimethyl sulphoxide (DMSO) was purchased from Merck (Darmstadt, Germany). Phosphoric acid and methanol were purchased from J. T. Baker (Phillipsburg, NJ, USA). Brain heart infusion agar (BHI agar) was purchased from Oxoid (Basingstoke, Hampshire, UK). Mueller–Hinton broth was purchased from BD (Sparks, MD, USA). Other chemicals and reagents were of analytical grade. All analyses were performed using ultra-purified water (Millipore, Illkirch-Graffenstaden, France).

The porcine eyes used in the in vitro experiments came indistinctively from males and females bred for human consumption (hybrids of Landrace and Large White) and slaughtered with 20 weeks old with approximately 100 kg. Eyes were collected immediately after the animals were slaughtered (Frigorífico Sabugy Ltda, Brasília, DF, Brazil), and were kept at 4 °C while transported to the laboratory.

### Drug quantification

Besifloxacin was quantified using an HPLC (LC-20CE—Shimadzu, Kyoto, Japan) equipped with a diode array detector (SPD-M20A) set at 340 nm and using a reversed-phase C_8_ column (Agilent ZORBAX EclipseXDB-C8—5 μm, 150 × 4.6 mm). The mobile phase consisted of 0.01% phosphoric acid: methanol (60:40 v/v) flowed in an isocratic mode at 1.2 mL/min. The column was maintained at 45 °C, and the injection volume of the samples was fixed at 10 μL. The method was validated according to ICH guidelines, being linear in the concentration range of 6.0–60.0 µg/mL (r = 0.9999; y = 13454x – 7208.20). The limit of detection (LOD) and limit of quantitation (LOQ) was found as 1 and 2 µg/mL, respectively. Intra-and inter-day precision of the method showed a coefficient of variation values not greater than 5%. The selectivity of the method has been previously evaluated against interferents (corneal, vitreous humor and aqueous humor homogenate) and interference was observed neither in besifloxacin concentration nor in the retention time of the drug. The recovery efficiency of the drug was 90.6 ± 5.9% for cornea, 85.6 ± 11.2% for vitreous humor, and 93.9 ± 3.1% for aqueous humor.

### Preparation of liposomal formulations

A series of conditions were tested to obtain the highest drug recovery and encapsulated drug amounts.

#### Drug solubilization medium

First, liposomes were obtained by the lipid film hydration technique^21^. Besifloxacin (2 mg) was solubilized either in the lipid phase, PC solution in chloroform (140 mmol/L), or in the aqueous phase, pH 7.4 HEPES buffer (25 mmol/L) containing NaCl (133 mmol/L)^28,29^ (originating Formulations F1 and F2, Table 1).

**Table 1:** Composition and characterization data of besifloxacin liposomes produced by lipid film hydration.

Formulation	PC (mmol/L)	CH (mmol/L)	SA (mmol/L)	SPM (mmol/L)	Drug (mg)	Drug solubilization phase	DR (%)	EE (%)
**Drug solubilization medium**								
F1	140	–	–	–	2	Lipid phase	63 ± 1.9	26 ± 5.18
F2	140	–	–	–	2	Aqueous phase	83 ± 1.5	37 ± 0.94
**pH of solubilization medium**								
LP PC	140	–	–	–	2	Aqueous acid phase	93 ± 0.5	51 ± 1.92
**Drug loading dose**								
F4	140	–	–	–	1	Aqueous acid phase	97 ± 0.8	52 ± 1.03
F5	140	–	–	–	3	Aqueous acid phase	83 ± 0.5	41 ± 2.19
F6	140	–	–	–	4	Aqueous acid phase	74 ± 1.0	45 ± 1.35
**Phospholipids concentration and cholesterol addition**								
F7	200	–	–	–	2	Aqueous acid phase	59 ± 0.9	55 ± 1.48
F8	40	10	–	–	2	Aqueous acid phase	56 ± 2.9	27 ± 8.86
F9	30	20	–	–	2	Aqueous acid phase	40 ± 1.9	30 ± 12.74
F10	20	10	–	–	2	Aqueous acid phase	60 ± 3.8	28 ± 12.19
**Cationic amines addition**								
F11	150	–	–	50	2	Aqueous basic phase	53 ± 0.8	21 ± 5.60
LP PC: SPM	150	–	–	90	2	Aqueous basic phase	68 ± 2.5	63 ± 1.86
F13	150	–	50	–	2	Aqueous basic phase	48 ± 1.3	13 ± 7.74
F14	150	–	90	–	2	Aqueous basic phase	65 ± 3.4	25 ± 0.66

*DR* drug recovery, *EE%* entrapment efficiency, *PC* phosphatidylcholine, *CH* cholesterol, *SA* sterilamine, *SPM* spermine.

The lipid film was formed by roto evaporation (Quimis, 344B2, São Paulo, Brazil), and rehydrated with 4 mL of the pH 7.4 HEPES buffer. To reduce the liposomes diameter, a 10-cycle extrusion process was performed through 200-nm-pore polycarbonate membrane using compressed nitrogen (Lipex, Northern Lipids Inc., Burnaby, B.C., Canada).

#### pH of solubilization medium

As a higher besifloxacin recovery was obtained when the drug was added in the aqueous phase, further experiments employed 1 mL of phosphoric acid at 0.01% (acid solution, pH 3) for drug solubilization and film hydration, followed by the addition of 3 mL of pH 7.4 HEPES buffer (originating Formulation LP PC, Table 1).

#### Drug loading dose

Optimal drug loading dose was evaluated by incorporating different drug amounts (1, 3, and 4 mg), following the same preparation method described above (originating Formulations F4 to F6, Table 1).

#### Phospholipids concentration and cholesterol addition

Different lipid stock-solutions of PC and CH solubilized in chloroform were also used at different concentrations (PC at 140 or 200 mmol/L^21,30^, being PC: CH molar ratios 4:1, 3:2, and 2:1) (originating Formulations F7 to F10, Table 1).

#### Cationic amines addition

Two cationic amines were evaluated as positive charge additives, SPM and SA. They were incorporated in lipid stock-solutions at PC: amine molar ratios of 3:1 and 5:3. Drug was solubilized in pH 11.6 HEPES solution alkalinized with NaOH (basic solution) and 1 mL of this solution was added to the dried lipid film. Lipidic film hydration was followed by the addition of 3 mL of pH 7.4 HEPES buffer (originating Formulations F11, LP PC:SPM, F13 and F14, Table 1).

Two formulations, LP PC and LP PC:SPM, demonstrated better drug recovery and encapsulation characteristics and were chosen for further experiments.

### Characterization of besifloxacin loaded liposomes

Hydrodynamic diameter and polydispersity index (PdI) were determined by dynamic light scattering and zeta potential by electrophoretic mobility (Zetasizer Nanoseries, Malvern Instruments, Worcestershire, UK) using the samples diluted in purified water (1:100 v/v) at 25 °C.

For calculating the EE, 500 μL of each besifloxacin liposome dispersion was filtered using a VivaSpin filtration tube (10,000 MWCO, Millipore, Burlington, MA, USA), under centrifugation for 25 min at 4000 rpm (Force G = 2150). Filtration parameters were validated allowing for 99.98 ± 2.32% of drug recovery in the filtrate (n = 3). The filtrate was diluted (1:20 v/v) in phosphoric acid 0.01 mol/L and quantified by high-performance liquid chromatography (HPLC) (“In vitro passive ocular drug permeation with simulated lacrimal flow” section) to determine the free-drug (FD) proportion. Total drug (TD) was determined by diluting each liposomal formulation in a surfactant solution (1% Triton X-100 solubilized in phosphoric acid 0.01 mol/L). Dilution factor was 1:20 v/v. Diluted formulation was then vortex stirred for 60 s to provide the rupture of the liposomes. Then, EE was obtained according to Eq. (1).

1 $$EE\left(\%\right)=\frac{\left(TD-FD\right)}{TD}\times 100$$

Drug recovery (DR) was obtained relating the total besifloxacin in liposomal dispersion with the amount of drug added (AD) at the beginning of the process, according to Eq. (2).

2 $$DR\left(\%\right)= \frac{TD}{AD}\times 100$$

Morphological analyses were performed using a transmission electron microscope (TEM; JEM 1011 Transmission Electron Microscope, JEOL, Tokyo, Japan – 100 kV). Images were captured with a GATAN BioScan camera (model 820, GATAN, PA, USA) using the Digital Micrograph 3.6.5 software (GATAN, PA, USA), samples were prepared as previously reported by de Sá et al.^21^. Briefly, to prepare the samples, diluted aliquots were deposited on a Formvar-coated copper grid (Electron Microscopy Sciences, PA, USA) air-dried for 10 min, and the excess of formulations was absolved by filter paper. After that, uranyl acetate solution 3% (w/v) (3 μL) was added and air-dried for 10 min, and the excess was also removed with filter paper.

Mucoadhesiveness was evaluated in vitro^31^. Briefly, mucin type III was hydrated with ultrapure water (1:10 w/v) overnight. Subsequently, it was diluted in pH 7.4 HEPES buffer to obtain a final mucin concentration of 1% (w/v). The dispersion was sonicated (Sonics Vibra Cell, VCX 500, Newtown, CT, USA) for 10 min with 25 Hz amplitude, pulse on for 30 s and off for 30 s to obtain particles’ sizes less than 700 nm. LP PC and LP PC: SPM were incorporated separately into mucin particles dispersion at a ratio of 1:1 (v/v) and then vortexed for 1 min. Soon after, samples were analyzed for their hydrodynamic diameter, as described before.

In vitro drug release profiles from LP PC and LP PC:SPM were determined using cellulose membranes mounted in Franz-type diffusion cells (diffusion area of 1.32 cm^2^). All tested formulations and the control contained 500 µg/mL of besifloxacin. The donor compartment was filled with 400 µL of each formulation while the acceptor compartment contained 15 mL of the pH 7.4 HEPES buffer. The system was maintained under magnetic stirring (600 rpm). Samples (1 mL) were withdrawn from the acceptor solution in 10, 20, 30, 60, and 120 min, and taken to drug quantification, while the same volume of “fresh” buffer was replaced to the acceptor compartment.

### Stability of formulations in stock and in the presence of electric current

Aliquots of the liposomes’ samples were stored at 6 °C and analyzed at 1, 7, 14, and 30 days for their visual aspects, mean hydrodynamic diameters, PdI, zeta potential, and EE to access their stability.

According to method described by Gelfuso et al.^11^, the electrical stability of the drug was also evaluated by subjecting the commercial formulation (Besivance 0.6%) to an electric current of 2 mA for 30 min, which was provided by the direct insertion of silver (+) and silver chloride (−) electrodes connected to a power source (Keithley Instruments, Keithley, OH, USA). The commercial formulation was previously diluted in pH 7.4 HEPES buffer to achieve a final concentration of 500 µg/mL. Samples were collected before and after 30 min of the current application for drug quantification.

Similarly, the electrical stability of liposomal formulations was evaluated by subjecting LP PC and LP PC: SPM to a 2 mA current for 30 min. Samples were also collected at 0 and 30 min and evaluated for hydrodynamic diameter, PdI, zeta potential, DR, and EE. Analyzes were performed in triplicate for each formulation. Electron paramagnetic resonance (EPR) spectroscopy studies were also performed after the experiment completion, as described below.

### Differential scanning calorimetry (DSC)

The individual components of the liposomes and their mixture were analyzed by a DSC-60 (Shimadzu, Japan) in a nitrogen atmosphere (50 mL/min) at a heating rate of 10 °C/min from 25 to 200 °C using 2–4 mg samples placed in platinum pans. The mixture of the components was performed in equal proportion in weight in order to maximize the interaction between them^32^.

### Electron paramagnetic resonance (EPR) spectroscopy

Liposomal formulations were incubated in tubes containing 1 μL of the spin labels 5-DSA or TEMPO (both diluted into ethanol at 5 mg/mL) at room temperature under moderate agitation for 30 min. The labeled samples were introduced in capillary tubes, which were sealed using a flame. Conventional EPR spectra were obtained with a Bruker EMX spectrometer (Rheinstetten, Germany), with temperature-control accessories, operating in the X-band (approximately 9.4 GHz) and the following instrumental parameters: microwave power = 2.0 mW; modulation frequency = 100 kHz; modulation amplitude of 1.0 G for 5-DSA and 0.3 G for TEMPO spin labels; magnetic field scan of 100 G for 5-DSA and 50 G for TEMPO spin probes; scan time = 168 s, and detection time constant = 41 ms. All EPR measurements were performed in triplicate at 25 °C and 32 °C (temperature at the ocular surface^33–35^) using a nitrogen stream system (Bruker, Rheinstetten, Germany).

### Ocular tolerance test (HET-CAM)

Ocular tolerability was assessed using the hen’s egg test-chorioallantoic membrane (HET-CAM) test, as previously reported^36^. Briefly, fertilized hen’s eggs were purchased from a poultry farm and used on the 10th day of fertilization. The equatorial side was placed up, and a 2 × 2 cm window was opened to expose CAM’s surface. Aliquots of tested formulations (300 µL) were placed directly on the exposed membrane. After 30 s, the CAM was carefully washed with PBS to ensure the total removal of the tested formulation. The CAM was visually observed for 5 min to detect the appearance of any of hyperemia or hemorrhage. PBS and NaOH solution at 1 mol/L were used as the negative and positive controls, respectively.

### In vitro iontophoretic drug permeation through the porcine cornea

Iontophoretic experiments were performed trough excised porcine corneas. Corneas were separated from other ocular structures and immediately mounted in Franz-modified diffusion cells (diffusion area of 1.32 cm^2^). In order to fit the concave cornea appropriately in the cell, the opening intended for the cornea placement had its edges raised by 2 mm. The acceptor compartment of the cells was filled with 15 mL of the pH 7.4 HEPES buffer and maintained under magnetic stirring (600 rpm). The donor compartment was filled with 400 µL of LP PC, LP PC:SPM, or the commercial formulation (Besivance 0.6%) used as control. Control formulations were diluted in HEPES buffer pH 7.4, whether necessary, to contain 500 µg/mL of besifloxacin. A salt bridge (3% agarose in 0.1 mol/L NaCl) connected the donor formulation to the positive electrode compartment (silver electrode immersed in saline solution). The negative electrodes (silver chloride) were immersed in the acceptor compartment of the diffusion cells. The electrodes were connected to a power source (Keithley Instruments, Keithley, OH, USA), which delivered a current density of 2 mA/cm^2^ for either 10 or 30 min. The same protocol but without the current application was used to evaluate the drug’s passive permeation from each formulation. Such experiments were performed in static condition, without fluid flow in the donor compartment. After 30 min of the experiment, samples of 1 mL were withdrawn from the acceptor solution, taken to drug quantification. Corneas were removed from the diffusion cells, washed with ultrapure water, cut in small pieces, added in 2 mL of phosphoric acid 0.01%, and submitted to 30 min of ultrasound bath. Samples were then filtered in nylon membranes and analyzed by HPLC for determining the drug content.

### In vitro passive ocular drug permeation with simulated lacrimal flow

**Figure 1:**
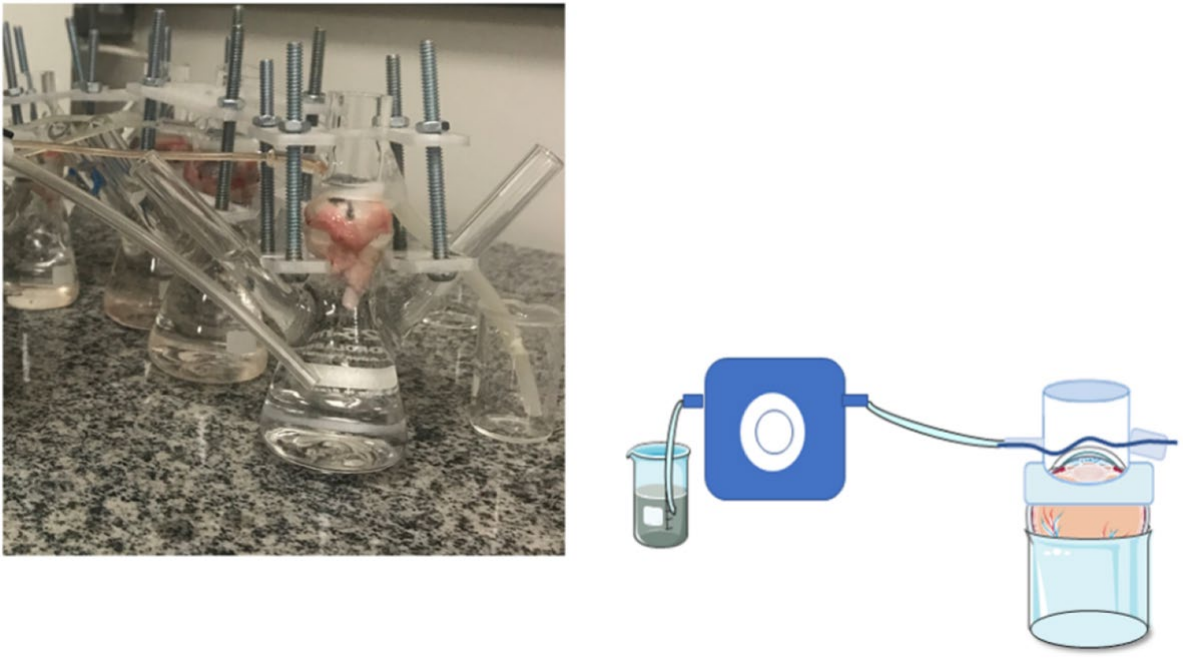
In vitro ocular model that simulates the lacrimal flow, maintaining a 20 µL/min-flow of PBS through a peristaltic pump.

In vitro permeation experiments with simulated lacrimal flow employed the entire porcine ocular globe, but only the cornea had immediate contact with the donor formulation (Fig. 1). The ocular globe was accommodated in a custom-made holder, and in this experiment, the acceptor compartment was not sampled. The donor compartment cell was designed and built to be connected with a peristaltic pump (MINIPLUS evolution, Gilson, Middleton, USA), maintaining a 20 µL/min-flow of isotonic PBS to mimic the tear flow^37^. Customized clips were obtained using a 3D printer, which keept the three structures connected (donor compartment, eyeball and acceptor compartment) (Fig. 1). Inlet was inserted 0.5 cm over the eye surface and outlet was inserted near the base (0.2 cm), inclined and with a larger internal diameter, so the drainage was controlled by the volume of the inlet and liquid would not accumulate. After the ocular globe and all holder compartments were assembled, tubing was connected, and the donor compartment was filled with 400 µL of LP PC, LP PC:SPM, or the commercial formulation (Besivance 0.6%) used as control. Control formulations were diluted in HEPES buffer pH 7.4, to contain 500 µg/mL of besifloxacin. Immediately after formulation instillation, the peristaltic pump was turned on, and the flow initiated. This parameter was scaled up by a factor 15 concerning previously reported tear production rate (1.3 µL/mL)^37^. At the end of the permeation experiment, the ocular globe was removed; aqueous and vitreous humor were sampled, and the cornea removed. Aqueous humor was collected with an insulin syringe, perpendicular inserted through the cornea. Vitreous humor was collected using a 5 mL syringe after cutting the eye ball with a scalpel. Drug extraction from the cornea followed the aforementioned protocol (item 2.7).

### Determination of minimum inhibitory (MIC) and bactericidal concentrations (MBC)

The MIC and MBC of the liposomes with besifloxacin against *Staphylococcus epidermidis* ATCC 14,990 and *Pseudomonas aeruginosa* ATCC 2327 were determined by the microdilution method^38^. Briefly, each inoculum, prepared from 24 h old cultures of *S. epidermidis* and *P. aeruginosa*, has grown on BHI agar. The colonies were suspended in PBS (0.5 McFarland—determined using a spectrophotometer at 625 nm) and diluted in Mueller–Hinton broth to achieve 2.5 × 10^6^ CFU/mL. This inoculum was added to a microplate containing dilutions of (a) free-besifloxacin, diluted in a pH 11 HEPES solution; (b) liposome with besifloxacin (LP PC); (c) positively charged liposome with besifloxacin (LP PC:SPM); and (d) the commercial formulation (Besivance). The final concentration of the bacterial inoculum in each well was 5 × 10^5^ CFU/mL, and the final concentration range of all treatments was 2.0 × 10^1^ to 9.0 × 10^–3^ μg/mL. Strains of *S. epidermidis* and *P. aeruginosa* in the absence of any antimicrobial were used as viability control, whereas wells containing only Mueller–Hinton medium were used as sterility control. Liposomes without drug were tested as vehicle control as well as the pH 11 HEPES solution. The microplates were incubated for 24 h at 37 °C. Bacterial growth was assessed using 0.01% resazurin solution. MIC was defined as the lowest drug amount that resulted in clear visualization of bacterial growth inhibition. To determine MBC, 5 μL aliquots of bacteria from each well, which does not show any bacterial growth, were cultured on BHI agar for 24 h, at 37 °C. MBC was the lowest concentration that allowed no visible bacterial growth on agar. All the assays were performed in triplicate.

### Statistical analyses

All data were expressed as mean ± standard deviation of at least three replicates. Statistical significance of the data was evaluated either by analysis of the variance (ANOVA one-way) with Tukey’s post hoc test or Student’s t-test with the level of significance fixed at 0.05 (GraphPad Prism version 6.00, La Jolla California, USA).

## Results

### Preparation of liposomal formulations

Components and concentrations were varied in order to obtain liposomes with the highest possible rates of DR and EE. Based on the results presented in Table 1, formulations LP PC and LP PC: SPM were chosen to continue the experiments.

Formulation LP PC, which incorporated the drug solubilized in acid solution (pH 3), employed a lipid:drug ratio of 300.6: 1 and achieved a high DR% of 93 ± 0.5% (meaning a final besifloxacin concentration of 467.84 ± 2.37 µg/mL) and a EE% of more than 50%. Final LP PC formulation pH was 6.79.

Formulation LP PC:SPM employed a lipid to drug ratio of 322.5:1 and, on the contrary, incorporated the drug solubilized in basic solution (pH 11.6) and obtained a lower DR % (68 ± 2.5) (meaning a final besofloxacin concentration of 335.81 ± 8.28 µg/mL) but a higher EE% (63.00 ± 1.86%). Final LP PC: SPM formulation pH was 9.70.

The mean hydrodynamic diameters were 177.2 ± 2.7 nm for LP PC, and 175.4 ± 1.9 nm for LP PC: SPM, with respective PdIs of 0.02 ± 0.01 and 0.071 ± 0.032. LP PC presented a residual charge of—5.7 ± 0.3 mV, while the LP PC:SPM were positively charged (zeta potential = 19.5 ± 1.0 mV).

**Figure 2:**
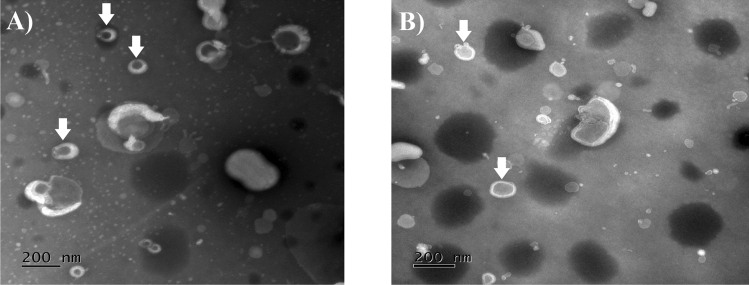
TEM images of (**A**) LP PC and (**B**) LP PC: SPM, indicated by white arrows (magnifications of ×80,000).

Figure 2 shows the morphological characterization of lipid nanosystems based on TEM images, where LP PC are presented as Fig. 2A and LP PC: SPM as Fig. 2B. Microscopy images clearly showed univesicular liposomal systems of approximately 200 nm.

**Figure 3:**
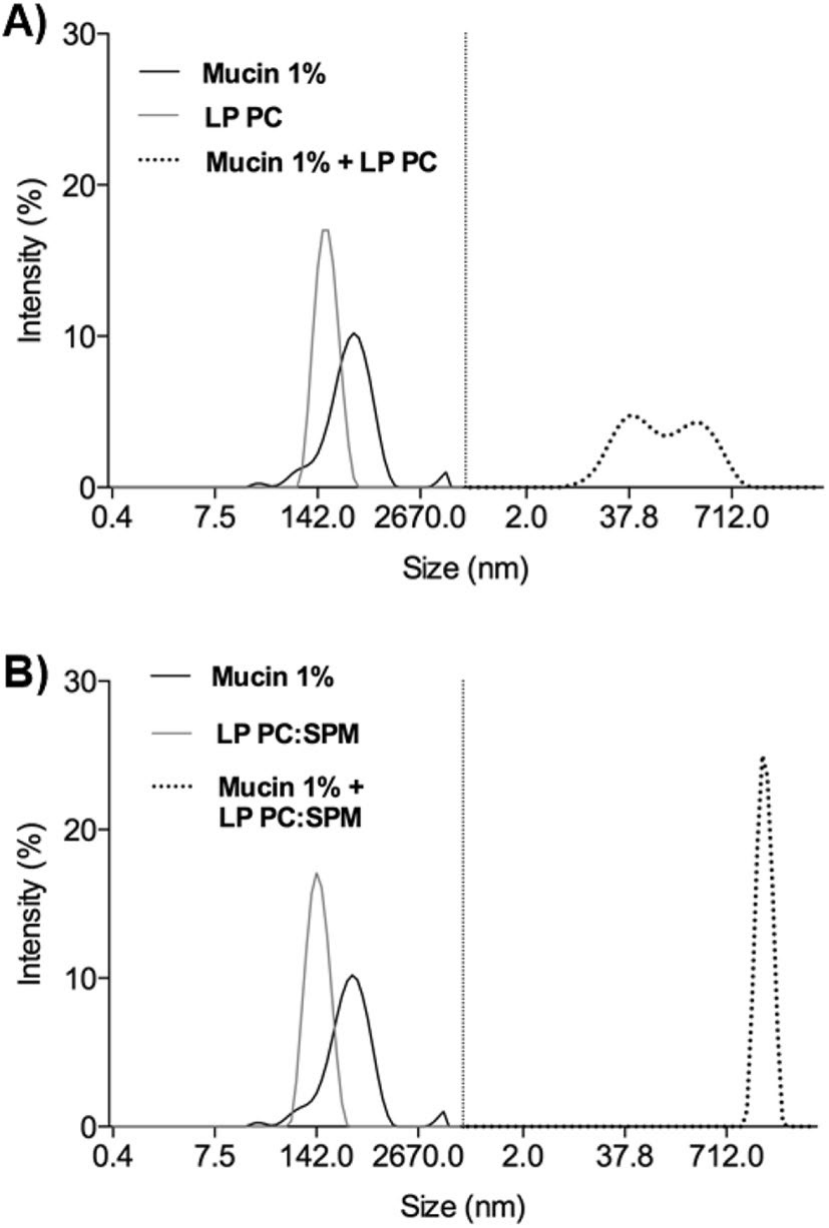
Mucoadhesive effect of the formulations LP PC (**A**) and LP PC: SPM (**B**) by hydrodynamic diameter variation of mucin 1% incorporation (t = 5 min). The grey line represents the hydrodynamic diameter of the liposome, the black, mucin 1%, and the dashed, the mixture of the liposome and mucin 1%.

Due to their positive charge residual, LP PC:SPM showed mucoadhesion characteristics. This can be noticed in Fig. 3, which shows a significant increase in hydrodynamic diameter of the formulation LP PC: SPM mixed with mucin particles due to agglomeration and junction of mucin to the liposome, but not for the LP PC formulation, which did not have charge additives.

Both liposomal formulations controlled the amount of drug released. There was no difference between the amount of drug released from either LP PC or LP PC:SPM (*p* > 0.05). After 10 and 30 min of experiment, respectively 5.59 ± 1.05 and 9.10 ± 1.29% of besofloxacin were released from LP PC and 5.50 ± 0.69 and 8.49 ± 1.06% respectively, were released from LP PC:SPM. Data values were close to LOQ of the analytical method (Supplementary Figure 1).

### Stability of formulations in stock and the presence of electric current

**Figure 4:**
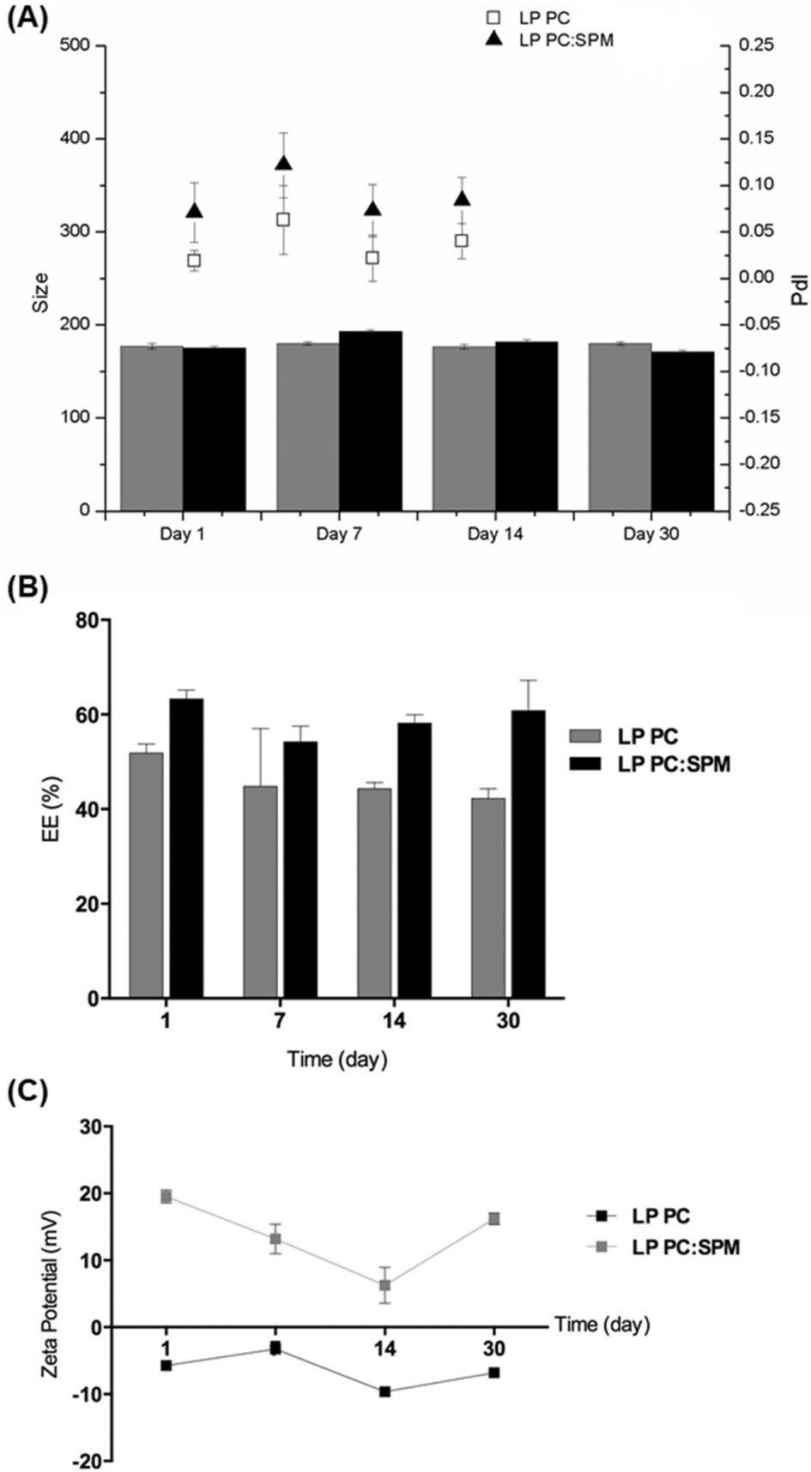
Stability of the liposomes’ formulations. (**A**) hydrodynamic diameter and PdI, (**B**) encapsulation efficiency (EE), and (**C**) zeta potential of LP PC and LP PC: SPM stored at 6ºC for 30 days.

We observed no significant changes (*p* > 0.05, ANOVA) in hydrodynamic diameter, PdI, and drug EE of the two formulations after 30 days; the samples were stored at 6 °C. The zeta potential presented significant changes (*p* ≤ 0.05, ANOVA) only in LP PC: SPM. These results indicate the physicochemical stability of the liposomes’ formulations (Fig. 4).

The stability of the drug in solution and entrapped in liposome formulations were also evaluated under a 2 mA-electric current application for 30 min, and data are presented in Table 2.

**Table 2:** LP PC and LP PC: SPM hydrodynamic diameter, polydispersion index (PdI), zeta potential, encapsulation efficiency (EE), and drug recovery (DR) before and after a 2 mA-electric current application (n = 3).

Formulations	Time (min)	Hydrodynamic diameter (nm)	PdI	Zeta potential (mV)	EE (%)	DR (%)
Besifloxacin solution	0	–	–	–	–	100.0
	30	–	–	–	–	95.3 ± 0.6
LP PC	0	177.2 ± 2.6	0.019 ± 0.011	− 5.7 ± 0.3**	51.8 ± 1.9*	100.0
	30	178.6 ± 8.9	0.034 ± 0.020	− 1.4 ± 0.1**	42.2 ± 2.0*	105.6 ± 2.3
LP PC:SPM	0	175.4 ± 1.9	0.071 ± 0.032	19.5 ± 1.0**	63.3 ± 1.8	100.0
	30	174.1 ± 2.2	0.075 ± 0.021	24.3 ± 2.5**	60.8 ± 6.4	99.9 ± 1.3

*(*p* ≤ 0.05, ANOVA),**(*p* ≤ 0.01, ANOVA).

No significant variation in hydrodynamic diameter and PdI (*p* > 0.05, ANOVA) of the liposomes formulations was observed with the application of iontophoresis. A statistically significant variation (*p* ≤ 0.01, ANOVA) in zeta potential, however, was noticed in both liposomes, most likely due to the silver ions released from the electrodes during the current passage.

In terms of entrapped drug, there was a significant (*p* ≤ 0.05, ANOVA) but subtle variation only in the LP PC, probably caused by a small disturbance in the liposomal structure with the electric current passage. Also, the total besifloxacin content (DR) was not significantly altered in any of the tested formulations (*p* > 0.05, ANOVA), proving the drug’s stability against the electric current.

### Ocular tolerance test (HET-CAM)

**Figure 5:**
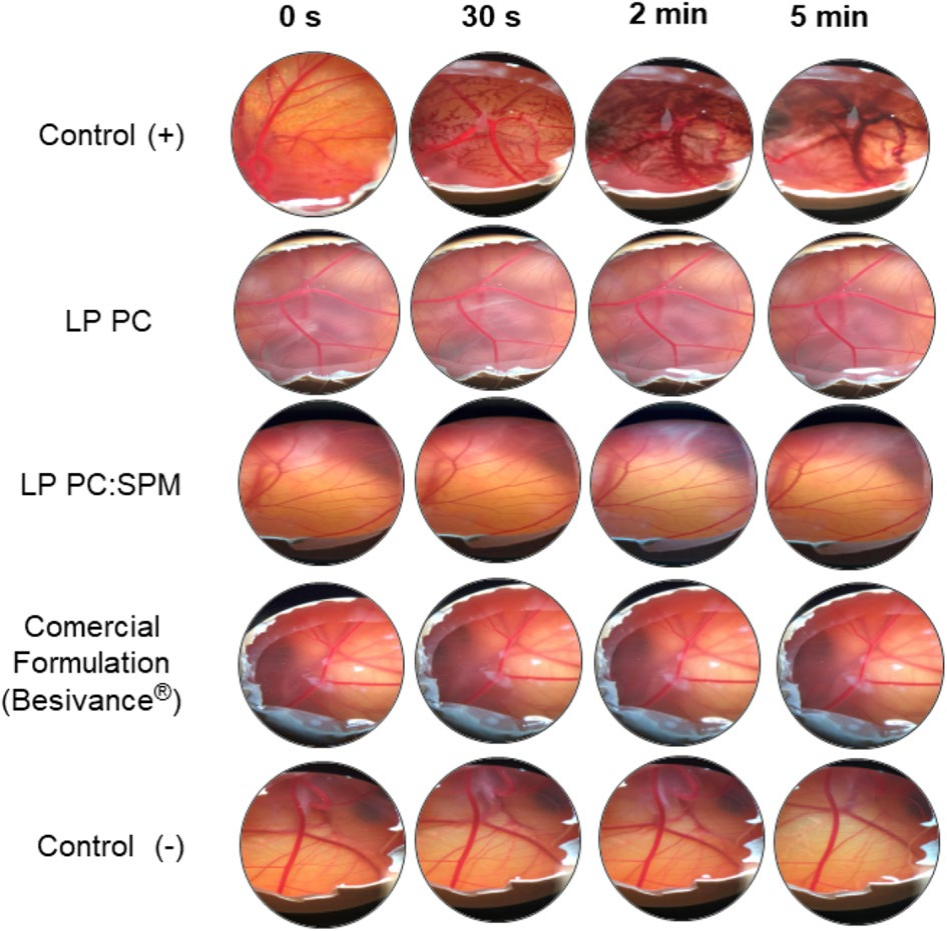
Irritability evaluation of LP PC, LP PC: SPM, and commercial formulation (Besivance 0.6%) in egg chorioallantoic membrane (CAM) compared to the positive control (+) using sodium hydroxide solution (NaOH) (1 mol/L) and negative control (−) using PBS.

Figure 5 presents pictures of CAM treated for 5 min with the liposomes’ formulations, commercial formulation, positive and negative controls. In contrast to the membrane treated with the highly corrosive positive control, which caused hyperemia and hemorrhage in the blood vessels, all tested formulations (LP PC, LP PC: SPM, and Besivance 0.6%) generated a response similar to the negative control (PBS) after 5 min of contact with the membrane, being classified as non-irritating.

### Characterization by differential scanning calorimetry (DSC) and electron paramagnetic resonance (EPR) spectroscopy measurements

**Figure 6:**
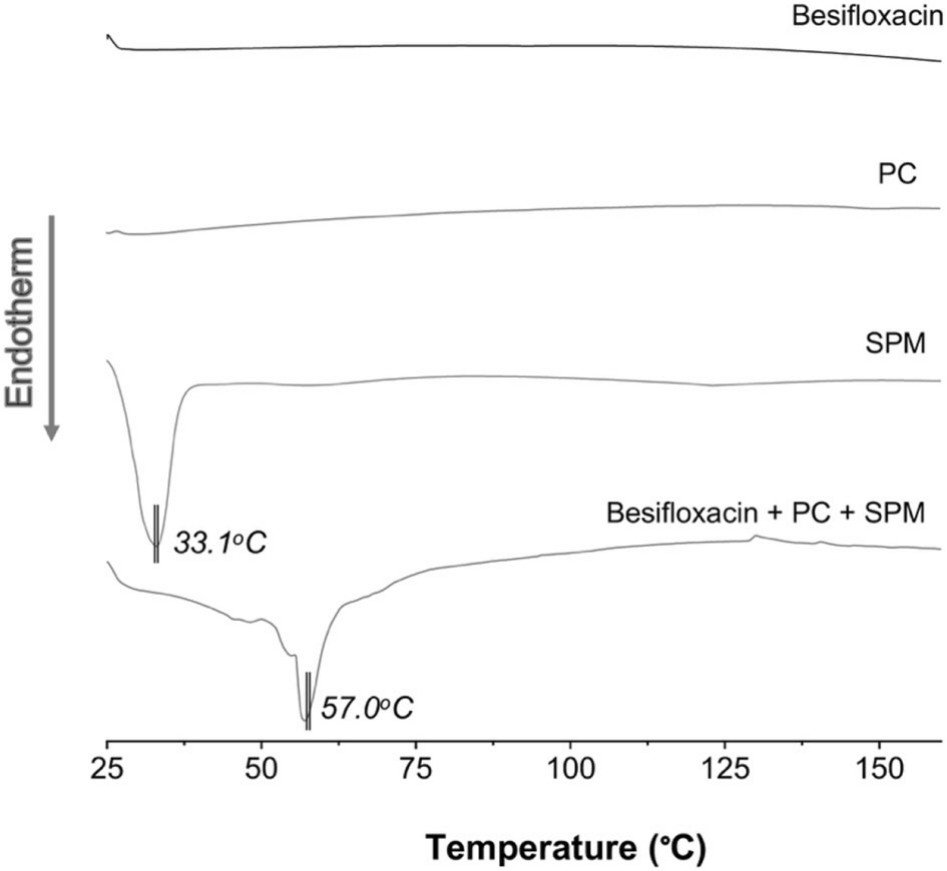
DSC curves of liposomes components as supplied, and their equimass mixture.

The DSC results (Fig. 6) showed the expected thermal profile for each component of the liposomes when analyzed individually. While besifloxacin and PC did not show thermal events in the studied temperature range, SPM melted at 33.1 °C. In turn, the mixture of liposome components revealed an interaction between them at low temperature, resulting in the displacement of the SPM melting peak to 57.0 °C. Still, such thermal interaction does not affect the stability of the system, which did not exhibit signs of thermal decomposition up to 150 °C.

**Figure 7:**
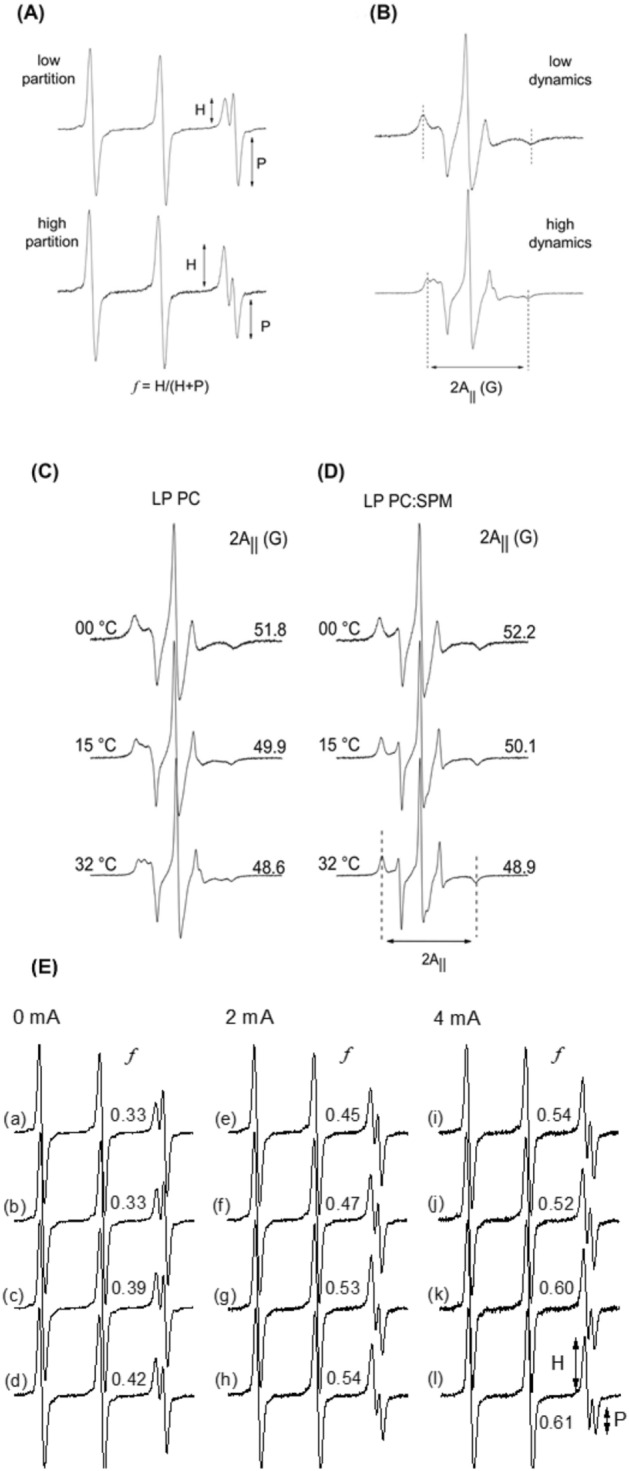
EPR spectra of liposomes labeled with the TEMPO (**A**) and 5-DSA (**B**) spin probes. The arrows (in spectra of **A**) and the dotted lines (in spectra of **B**) illustrate how the partitioning parameter (*f* = H/(H + P)) and the maximum hyperfine splitting parameter (2A_||_) were obtained from the experimental line. Large values of *f* indicate high partitioning, i.e., high spin label-membrane interaction and large values of 2A_||_ indicate more restricted molecular motion of the spin probe. EPR spectra of the 5-DSA spin label associated with (**C**) LP PC and (**D**) LP PC:SPM-besifloxacin liposomes at different temperatures. The values of the maximum hyperfine splitting parameter 2A_||_ are indicated for each spectrum (determined as indicated in the last spectrum). The experimental error associated with the 2A_||_ values is 0.5 G. The intensities of the experimental spectra (on the y-axis) are normalized, and the total magnetic field range is 100 G. (**E**) EPR spectra of different PC liposomes labeled with the TEMPO spin probe at 32 °C and exposed to 0, 2, and 4 mA of electric current intensities for 30 min. The values of the partitioning parameter (*f* = H/(H + P)) are indicated for each spectrum. The intensities of all spectra, represented on the y-axis, are normalized. The total magnetic field range is 50 G. The experimental error associated with the f determination is 0.3. Spectra (a), (e) and (i) refer to pure LP PC liposomes; spectra (b), (f) and (j) refer to LP PC-besifloxacin liposomes; spectra (c), (g) and (k) refer to LP PC:SPM liposomes; whereas spectra (d), (h) and (l) refer to LP PC:SPM-besifloxacin liposomes.

The EPR spectra of liposomes membranes labeled with TEMPO and 5-DSA spin probes are shown in Fig. 7A,B, respectively. The TEMPO spin label, commonly used to mimic small drugs in order to evaluate its interactions with lipid membranes^39,40^, is a small amphipathic probe able to partition between the lipid and the aqueous phases of many membranes, i.e., distributed into two spin label populations, one dissolved in the hydrophobic environment (component H) and one dissolved in the polar environment (component P)^40,41^. The relationship between these two components, represented by partitioning parameter *f* = H/(H + P), Fig. 7A, is an indicator of the drug (TEMPO) absorption. In fact, large values of *f* indicate high drug-membrane partitioning^39^, and the behavior of these parameter values may be used to monitor membrane-molecule interactions, membrane stability, and membrane dynamics. Alternatively, the 5-DSA, a spin probe derived from the stearic acid, was used to monitor the fluidity of liposomes membranes^41,42^. The dynamics of the 5-DSA, which is essentially stabilized in the membrane in the same way that the native lipids, can be assessed by the outer hyperfine splitting parameter (2A_||_), that can be obtained from the EPR spectrum as showed in Fig. 7B. For this spin label, large values of the 2A_||_ parameter, indicate a restricted molecular motion of the probe and a rigid lipid bilayer. In this work, these two parameters were used to characterize and to monitor the changes of the liposome lipid bilayers upon its composition and the presence of an electrical current.

Figure 7C,D show the EPR spectra of 5-DSA incorporated into LP PC and LP PC:SPM- besifloxacin liposomes at different temperatures, respectively. Note that the values obtained for hyperfine splitting parameter 2A_||_ indicated that there is no significant difference on the lipid fluidity between LP PC and LP PC:SPM-besifloxacin liposomes in the three temperatures studied, i.e., the presence of besifloxacin and SPM did not cause any significant changes in 2A_||_ values. The narrowing of the hyperfine lines of the 5-DSA spectra was observed upon temperature increase from 00 to 32 °C, as a result of increased lipid dynamic of liposomes membranes, which can be associated with a higher molecular disorder^43^.

The effect of electric current on lipid order and motion was also investigated by EPR spectroscopy using the two previously referred spin labels. Experimental spectra of different PC formulations labeled with TEMPO at 32 °C and undergone to 0, 2, and 4 mA of electric current intensities during 30 min are shown in Fig. 7E. As can be seen, for all lipid formulations, the partitioning of TEMPO was increased with increasing of electric current intensities, indicated by the changes in the values of the parameter *f*. That is, the application of an electric current up to 4 mA promoted a disorder on membrane-water interface favoring the permeation of TEMPO spin probe (increasing H component) compared to passive samples (without current application). Interestingly, this effect occurs only in the interfacial region and no changes were observed on the lipid bilayer fluidity as monitored by the 5-DSA label, i.e., the iontophoresis did not cause any significant changes in 2A_||_ values, even for electric current up to 4 mA during 60 min (as illustrated in Supplementary Figure 2). On the other hand, the values of parameter *f* were higher for LP PC:SPM-besifloxacin (d, h and l) and LP PC:SPM (c, g, and k) liposomes when compared to LP PC (a, e and i) and LP PC-besifloxacin (b, f, and j) liposomes even under passive conditions, corroborating the interaction with SPM identified by DSC. The incorporation of besifloxacin did not cause any significant changes in parameter *f* or 2A_||_ values, and no differences were observed when the formulations were subjected to 2 mA of electric current during 30 or 60 min as shown in Supplementary Figure 3.

### In vitro iontophoretic drug permeation through the porcine cornea

**Figure 8:**
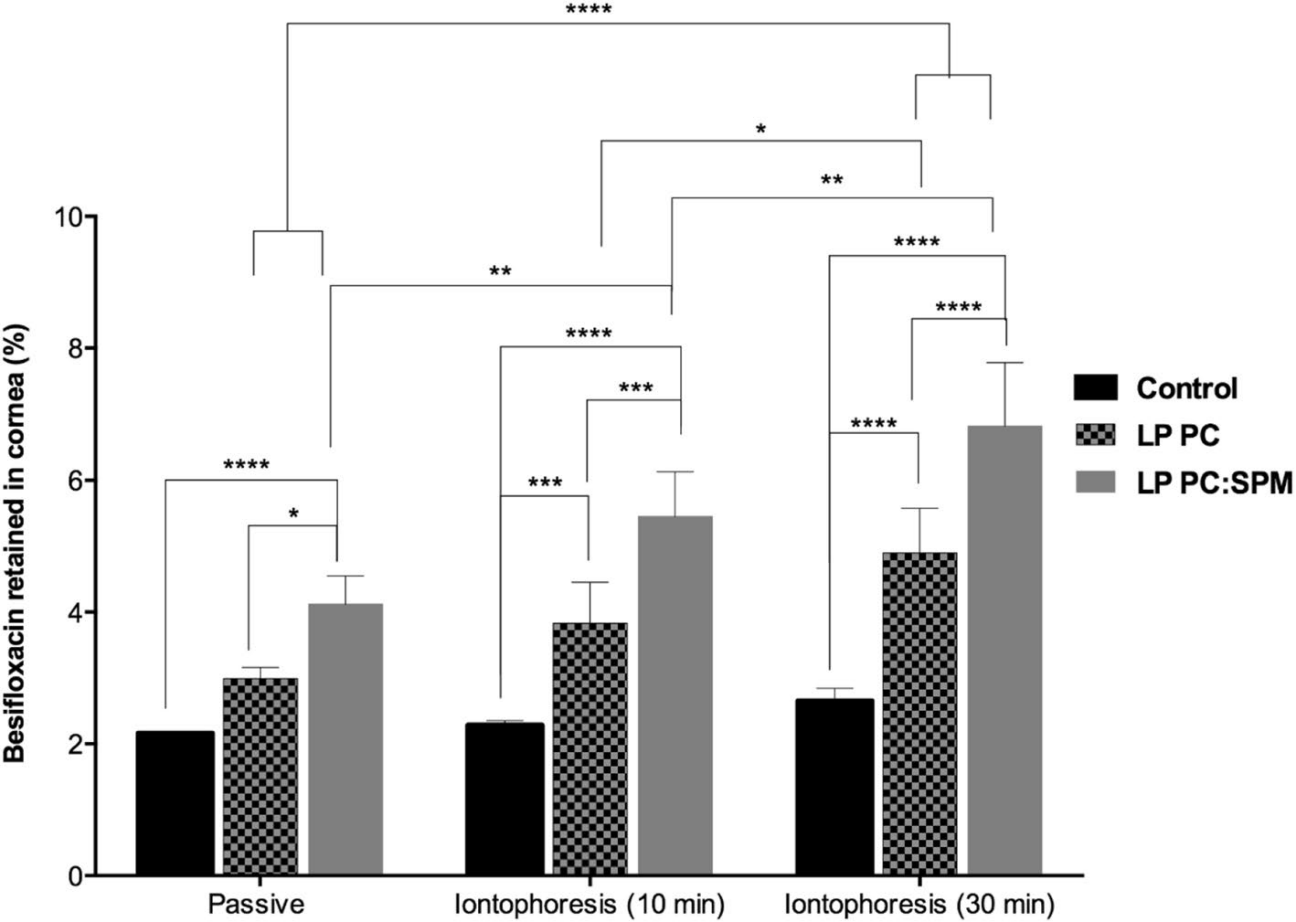
Besifloxacin retained in the cornea from LP PC, LP PC:SPM, and control (Besivance) after passive (30 min) or iontophoretic (10 or 30 min at 2 mA/cm^2^) permeation experiment trough excised porcine cornea (n = 4). ^*^(p ≤ 0.05, ANOVA); ^**^(p ≤ 0.01, ANOVA); ^***^(p ≤ 0.001, ANOVA); ^****^(p ≤ 0.0001, ANOVA).

The results from in vitro iontophoretic besifloxacin permeation through the porcine cornea for either 10 or 30 min under the influence of 2 mA/cm^2^, compared to the results of passive permeation for 30 min (without the electrical current), are presented in Fig. 8. According to different DR% of each liposomal formulation (Table 1), LP PC had a final drug concentration of 467.84 ± 2.37 µg/mL. while LP PC:SPM contained 335.81 ± 8.28 µg/mL of besifloxacin. Because of this difference, to better evaluate the delivery from each formulation, results are presented in terms of besifloxacin % retained in the cornea in relation to the applied drug amount. The commercial formulation Besivance was diluted in HEPES buffer pH 7.4 to achieve a final concentration of 500 µg/mL.

Under passive static application, LP PC:SPM formulation showed a significantly higher besifloxacin delivery compared to the commercial formulation Besivance (control) (*p* < 0.0001) and LP PC (*p* < 0.05), which, in turn, was not different to control (*p* > 0.05). The application of the electric current (2 mA/cm^2^) enhanced the amount of drug delivered from all formulations compared to the passive condition, but the enhancement was higher for the liposomal formulations compared to the control (*p* < 0.01) (Fig. 8). In absolute drug amounts, after 30 min of iontophoresis LP PC and LP PC:SPM formulations provided 6.95 ± 0.83 and 6.94 ± 0.83 µg/cm^2^, respectively, of besifloxacin retained in the cornea, representing an increase of approximately 65% compared to the passive condition. After the same 30 min of iontophoresis, the control formulation provided 4.02 ± 0.25 µg/cm^2^ of besifloxacin retained, meaning an increase of 23% compared to the passive condition. When the % of besifloxacin delivered from the applied formulation is analyzed (Fig. 8), a difference in delivery between each liposomal formulation (*p* < 0.05 in all conditions) is observed. While after 30 min of iontophoresis LP PC delivers 4.90 ± 0.67% of the applied drug dose, LP PC:SPM delivers 6.82 ± 0.96% (meaning a 40% higher delivery efficiency). Still, this was the same difference observed in the passive condition (2.99 ± 0.17 compared to 4.12 ± 0.43%, respectively). Hence, iontophoresis promoted a 65% increase on drug delivery independently from the liposomal formulation applied. No drug was quantified in the acceptor solution.

### In vitro passive ocular drug permeation with simulated lacrimal flow

When a simulated flow (≅ 20 µL/min) was applied in the donor compartment, diluting and draining applied donor formulation, the only liposomal formulation that provided a statistically significant higher drug penetration compared to the control was the positively charged LP PC:SPM (*p* < 0.002, t-test). LP PC:SPM provided 4.26 ± 0.74% of the applied drug amount retained in the cornea, while the control and the LP PC formulations provided, respectively 2.29 ± 0.15 and 2.65 ± 0.34%. Hence, the positively charged liposomal formulation had a significantly higher drug delivery than the LP PC formulation (*p* < 0.008). Also, no drug was found in the vitreous and aqueous humor after 30 min of the experiment.

### Determination of MIC and MBC

The results of MIC and MBC for *S. epidermidis* and *P. aeruginosa* are described in Table 3*.* Both MIC and MBC show the same results for all treatments for *S. epidermidis*. However, only Besivance, the commercial formulation, showed a higher MIC and MBC values than other treatments for *P. aeruginosa*. MIC and MBC are higher for *P. aeruginosa* than *S. epidermidis*. All controls were viable except for the control with only the culture medium (sterility control).

**Table 3:** Minimum inhibitory concentration (MIC) and minimum bactericidal concentration (MBC) of liposomes with besifloxacin against *S. epidermidis* and *P. aeruginosa* planktonic cells.

Treatments	*S. epidermidis*		*P. aeruginosa*	
	MIC (μg/mL)	MBC (μg/mL)	MIC (μg/mL)	MBC (μg/mL)
Besifloxacin	0.156 ± 0.0	0.156 ± 0.0	0.625 ± 0.0	0.625 ± 0.0
LP PC	0.156 ± 0.0	0.156 ± 0.0	0.625 ± 0.0	0.625 ± 0.0
LP PC:SPM	0.156 ± 0.0	0.156 ± 0.0	0.625 ± 0.0	0.625 ± 0.0
Besivance	0.156 ± 0.0	0.156 ± 0.0	1.25 ± 0.0	1.25 ± 0.0
LP PC vehicle control	All viable	All viable	All viable	All viable
LP PC:SPM vehicle control	All viable	All viable	All viable	All viable
pH 11 HEPES solution	All viable	All viable	All viable	All viable
Viability control	All viable	All viable	All viable	All viable
Sterility control	Inviable	Inviable	Inviable	Inviable

Data are expressed as mean ± standard deviation (n = 3). Treatments were besifloxacin in solution at 500 µg/mL; LP PC containing 467.84 ± 2.37 µg/mL of besifloxacin; LP PC:SPM containing 335.81 ± 8.28 µg/mL of besifloxacin; Besivance: commercial formulation diluted in HEPES buffer pH 7.4 to 500 µg/mL of the drug; LP PC vehicle control without drug; LP PC:SPM vehicle control without drug; pH 11 HEPES solution: vehicle control of the drug; Viability control: strains of *S. epidermidis* and *P. aeruginosa;* Sterility control: culture medium.

## Discussion

This study describes the production and characterization of two liposomal formulations to incorporate besifloxacin, named LP PC:SPM and LP PC. These systems were prepared respectively with or without spermine to confer or not a positive charge to the nanostructure.

Spermine is a biogenic polyamine that have a net positive charge at physiological pH^44^. Polyamines ophthalmic applications have been described in the literature. Gelatin nanoparticles containing spermine have shown adequate safety level for their application in the ocular surface using the XTT toxicity test^45^. Also, in vitro cytotoxicity, hemolysis, hemagglutination, genotoxicity, and oxidative stress and in vivo morphologic and physiologic cornea change evaluations show the good biocompatibility Quantum Dots synthesized by direct pyrolysis of spermidine, a similar polyamine^44^.

Liposomes prepared in this study had approximately 180 nm of hydrodynamic diameter, showing a very homogeneous size distribution, with PdI values below 0.2. Nanostructures of this order of magnitude have been shown to have their transport through the cornea facilitated, in addition to preventing eye irritation during the application or visual disturbances^46^.

Besifloxacin physicochemical characteristics make it a challenging molecule to be encapsulated in liposomes. The low water solubility at physiological pH (0.143 mg/mL) reduce the molecules tendency for accumulation in the aqueous nucleus, while its polar nature (Log P 0.7) hamper their interaction with the lipids constituting the lipidic membrane. Previous studies have reported the preparation method had great influence on the EE% of polar molecules^47^. Indeed, liposomes preparation using active drug loading method^30^, in which, the drug was incorporated during film hydration at a pH for the highest drug solubility, achieved EE greater than 50% for both formulations. Such a technique was carried out with adaptations, using an acid pH solution to form liposomes, then an external solution of basic pH and vice versa, producing liposomes with different pH inside and outside of the core. Because of this difference, the charged drugs diffuse across the membrane with more difficulty, which is an advantage for drug encapsulation^48,49^.

**Figure 9:**
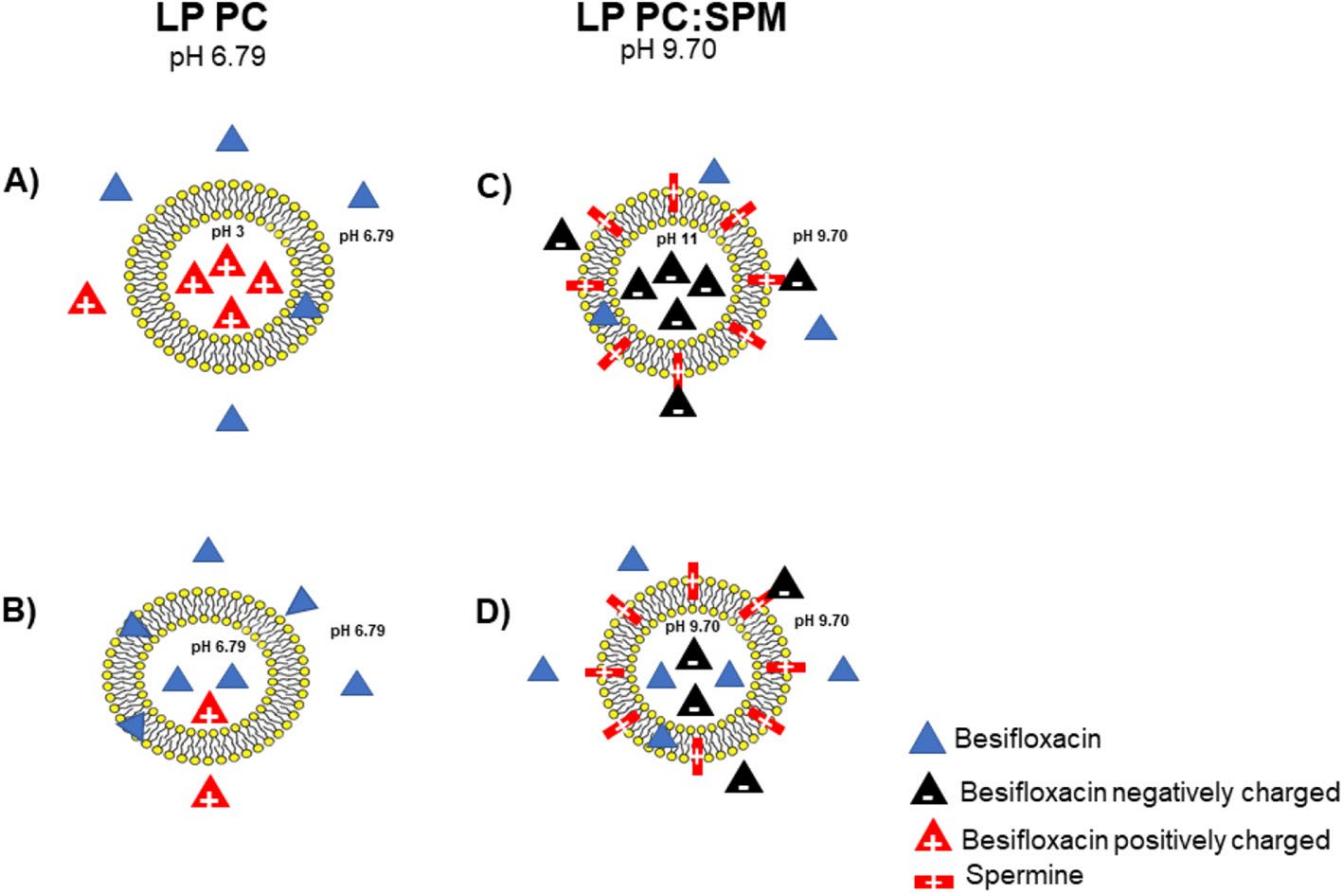
Scheme of possible dispositions of the besifloxacin in LP PC (**A**,**B**) and LP PC: SPM (**C**,**D**), according to the characteristics and the method of preparation of each liposome.

While besifloxacin has two pKa, strongest acid of 5.64 and strongest basic of 9.67, and considering that the technique of preparing the LP PC first involved solubilizing the lipid film at pH 3, followed by adjusting the pH to 6, two hypotheses are raised to explain the encapsulation of the drug in this case, as schematized in Fig. 9.

As formulation LP PC used an acidic solution (pH = 3) for besifloxacin solubilization and the first lipidic film hydration, which was then solubilized in neutral HEPES buffer, part of besifloxacin molecules might be entrapped at the liposome aqueous nucleus maintaining this lower pH value and, as a consequence, all molecules in the nucleus are positively charged. This hypothesis is depicted in Fig. 9A. In the first one (Fig. 9A), the liposome nucleus would maintain the acidic pH even after redispersion at pH 6.8. In this case, the drug, positively charged, would be trapped in that region. Alternatively, when the formulation is solubilized in the HEPES buffer, the whole formulation is neutralized, and the nucleus and medium share the same final pH (6.79). In this case, most of the besifloxacin molecules are neutral. This hypothesis is depicted in Fig. 9B. This alternative seems unlikely because the drug solubilization in acidic medium proved to provide a higher EE compared to direct drug incorporation in neutral buffer solution (51 ± 1.92 and 37 ± 0.94%, respectively, Table 1). Based on these EE values and the first hypothesis, more than 27% of the besifloxacin molecules are positively charged.

Accordingly, formulation LC PC:SPM used a basic solution (pH = 11.6) for drug solubilization. In this case, EE was even higher (63 ± 1.86%). When more spermine was employed (LP PC:SPM compared to F11**,** Table 1), the higher was the EE. At pH 11.6, besifloxacin is negatively charged (strong basic pKa = 9.91). Hence, the hypothesis here is negatively charged molecules are attracted by the small positive additives added to the liposomal formulation (explaining also the higher EE of LP PC:SPM compared to LP PC, Table 1, *p* > 0.05), and negatively charged besifloxacin molecules remain trapped at the aqueous nucleus and also between the bilayer lamella (Fig. 9C). Nonetheless, as in this case, the final formulation pH was 9.70, even if the nucleus has the same pH as the medium, a great part of besifloxacin molecules are still negatively charged (Fig. 9D).

The morphological characterization of lipid nanosystems is shown in Fig. 2A for LP PC and Fig. 2B for LP PC:SPM. Both systems presented as spherical unilamellar liposomes smaller than 200 nm. The lighter colored edges clearly indicate the formation of the lipid bilayer of the liposomes^50^.

Spermine, which we called positive additive, has two primaries and two secondary amino groups that interact with the hydrophilic portion of lipid nanosystems^27,51,52^. As expected, zeta potential results showed liposomes surface was positive with spermine insertion. In addition to the hypothesis of such charge influencing drug encapsulation, LC PC:SPM liposomes demonstrated higher mucoadhesive characteristic (Fig. 3B) compared to LP PC (Fig. 3A), which might promote liposomes adhesion when in contact the negative charge of the corneal surface, and potentially increase contact time with the cornea and decrease drug clearance by tear flow^7,53^.

Spermine addition, however, did not influence drug release from the formulation (Supplementary Figure 1). For the two hours of experiment drug release was the same (*p* > 0.05) from both formulations LP PC and LP PC:SPM, approximately 19%, showing a controlled release from the liposomes. Still, after liposome contact with the cornea an interaction of the bilayer liposomal membrane with the epithelial cells is expected to occur, releasing a more pronounced amount of drug^11^.

Both liposomal formulations showed to be stable under all conditions evaluated. LP PC and LP PC:SPM showed no statistically significant variations in hydrodynamic diameter, PdI, and EE after being stored at 6 °C for 30 days, only a significant change in the zeta potential of LP PC: SPM, indicating the formulations are physically stable (Fig. 4).

The entrapped drug in LP PC from the day of production to 30 days of storage varied from 51.8 ± 1.9% to 42.2 ± 2.0%, while in LP PC: SPM varied from 63.3 ± 1.8% to 60.8 ± 6.4%. These variations were not statistically significant according to Tukey’s post hoc test, but the lower variation in LP PC: SPM may be due to more rigidity provided by the insertion of spermine in liposomes. Similar behavior was observed when liposomes were submitted to an electric current of 2 mA for 30 min, where no relevant changes were observed in drug entrapped LP PC:SPM samples and a significant reduction (*p* ≤ 0.05) in besifloxacin entrapped in LP PC was observed (Table 2).

These results indicating a high liposomal stability were corroborated by the EPR spectroscopy measurements, in which the lipid dynamic behavior of liposome membranes and the effect of iontophoresis on the fluidity of these carriers were investigated by using 5-DSA and TEMPO spin labels with nitroxide group localized at different positions of the liposomes’ membranes.

The thermal behavior of the molecular motion of the 5-DSA spin probe structured in liposome membranes, after drug loading, is essential for designing stable liposomal formulations. Here, the physical stability with the thermal effect on PC formulations was evaluated in order to get a direct comparison of the besifloxacin incorporation. Our results showed that the increase in temperature induced increased mobility of liposomes lipids by providing the energy required to decrease the barrier that the spin probe must overcome to achieve higher states of motion, which can be seen by the reduction of 2A|| values^39,40,43^. Moreover, significant changes in the liposome membrane fluidity, associated for instance, with compromised nanocarrier structure, can be detected by EPR spectroscopy by drastic line-shape alterations of the 5-DSA spectra^43,54^. In this sense, the thermal effect observed here (up to 32 °C) induces only a subtle lipid reorganization, which leads to no evident alteration in the fluidity of the liposome’s membranes, retaining they structural stability. Thus, the temperature of 32 °C was deemed suitable for subsequent iontophoresis.

Curiously, the presence of besifloxacin or spermine seems not to change the dynamic of the spin probe, which is an important feature related to the stability of medicinal products like liposomes. This result can be related to the lower molar ratio of lipid:besifloxacin (1:0.1), unable to destabilize liposomal structure as monitored by the EPR spectroscopy. These results are following those reported by Matsingou and Demetzos that using DPPC lipid membranes after incorporating Labdanes, showed that the liposomes were stable at the molar ratio of approximately 1:0.1 (9:1) DPPC:Labdanes^55^. On the other hand, Dos Anjos et al.^54^ verified alterations in lipid fluidity of DPPC liposome incorporated with small terpenes such as 1.8-cineole at the molar ratio of 1:0.5 DPPC:terpenes, a very high concentration compared to the ratio of 1:0.1 lipid:besifloxacin used in our study.

Since iontophoresis acts by favoring drug partition in membranes systems, by energy-driven means^56^, the EPR spectroscopy is an excellent alternative to traditional techniques to assess the effect of electric current on permeability and stability of lipid systems. Our results showed iontophoresis could promote TEMPO migration from the aqueous to a hydrocarbon phase, i.e., increasing the partitioning of this spin label on liposomes. Interestingly, this effect was dependent on the magnitude of the electric current, being higher for LP PC:SPM-besifloxacin compared to the other formulations. However, even for electric current up to 4 mA for 60 min, no changes in lipid fluidity was detected, suggesting that the effect of electric current on the liposomes is local. Besides, in previous work, in which iontophoresis was applied in positively charged chitosan-coated liposomes, we have also observed that the electric current (2 mA for 30 min) did not change the stability of the formulations, but only altered the lipid rearrangement of PC liposomes by changing the nanocarriers superficial charge distribution^11^.

As mentioned in the introduction, one of the main advantages of a liposomal formulation for an ophthalmic application is the administration of insoluble molecules in a non-viscous formulation that avoids visual disturbances. Nevertheless, for an ophthalmic application, safety is an essential characteristic. HET-CAM is a qualitative and low sensitivity evaluation method for moderately irritating products that have been entirely used for evaluating eye irritability, alternatively to the use of animal models, which has currently been questioned^57,58^. The formulations here evaluated have a final pH of 6.79 for LP PC and 9.70 for LP PC:SPM within the range is considered non-irritant to the eye (6–10)^59^. HET-CAM tests showed that none of the liposomes were irritating (Fig. 5), which is suitable for the ocular application.

Additionally, to be more convenient to be administered, liposomes provided a higher drug accumulation within the cornea in all situations studied compared to the control formulation (Fig. 8). This was expected since liposomes grant a better interaction of the drug with the ocular surface, which might facilitate drug penetration^60–62^.

Ocular iontophoresis has already been studied for many years as a non-invasive drug delivery technique. According to targeted site there are two modalities of applications, transcorneal iontophoresis, which targets the cornea and, in general, the anterior segment of the eye, and transscleral iontophoresis, applied to the sclera, targeting the posterior segment^63^. As this paper is intended to deliver and antibiotic to the cornea, transcorneal iontophoresis was applied using and “eye cup” applicator. Similar devices have been described and had their safety evaluated in preclinical and clinical applications^13,64–67^ . For being a less resistive tissue than the skin, the cornea supports higher current densities, which enables the application for lower time periods. For example, current densities from 1.8 to 5.0 mA/cm^2^ for 5 min have been safely applied on human cornea^65,68^, while in vivo tolerance studies in rabbits confirmed the safety of 6.25 mA/cm^2^ current density^63^. Hence, ocular iontophoresis has notable clinical potential for being less invasive than other conventional clinical procedures, as epithelial debridement or intravitreal injections. Technical challenges and the need of application by a trained professional, may be viewed as a limitation.

As expected, iontophoresis increased drug penetration from all tested formulations, and the increase promoted was proportional to the time of technique application (Fig. 8). However, contrary to what was expected^17,63^, corneal penetration of the drug from the positively charged LP PC:SPM formulation was not further favored when compared to the neutral liposome (LP PC). As demonstrated in the results section, iontophoresis promoted a 65% increase on drug delivery independently from the liposomal formulation applied. Such results align with other assumptions that liposomes only enhance drug penetration by improving drug contact and interaction with biological membranes, but they do not penetrate the tissues as an entire rigid entity^11^. Therefore, multiple phenomena happen simultaneously. On the one hand, the positive liposomal charge enhances nanosystem electromigration towards the tissue, promoting drug-tissue interaction, and enhancing drug penetration. On the other hand, part of besifloxacin molecules, negatively charged, are attracted to the positive electrode and do not have enhanced electromigration towards the tissue^69,70^. Concurrently, free drug present in the formulation, corresponding to approximately 37% of the drug dose, penetrates the cornea due to the passive penetration component^12,63^.

The phenomenon occurring with the LC PC are also multiple. Even though liposomes are not charged, and the described nanosystem electromigration towards the tissue is not expected, within the formulation, there are positively charged besifloxacin molecules (Fig. 9), which might have much higher electromigration, considering anode iontophoresis was used, i.e., the formulation was added in contact with the positively charged electrode. Iontophoretic studies indeed show higher electromigration of positively charged molecules^71,72^. Therefore, the drug permeation enhancement provided by the liposomal added positive charge is equivalent to the enhancement provided by the drug charge. Thus, the positive charge of LP PC:SPM did not represent an advantage over LP PC to stimulate the passage of drug through the cornea by iontophoresis for this particular molecule.

Still, the positive charge of LC PC:SPM showed to provide an advantageous mucoadhesiveness that allowed the amount of drug recovered from being equal to the drug recovered in a static situation even when the formulation was challenged with a simulated tear flow. The results show LP PC did not present a significant difference in dynamic permeation compared to control; however, the significant variation remained for LP PC:SPM in these conditions compared to control. Thereby, the positively charged liposomal formulation presented a better performance than the LP PC formulation (*p* < 0.008). This means the added charge confers formulation resistance to drainage. Not only this, the positive charge additive also enhanced the drug delivery compared to the neutral liposomal formulation (LP PC) in both static (4.12 ± 0.43 and 2.99 ± 0.17%, *p* < 0.003, respectively) and dynamic conditions (4.26 ± 0.74 and 2.65 ± 0.34%, *p* < 0.008, respectively). Polyamines have been reported to interact with bacterial cell membranes, compromising their structure^44,73,74^. In this way, spermine may have acted as a permeation enhancer facilitating besifloxacin penetration into the cornea.

Another advantage of such mucoadhesiveness is the possibility of an intimate contact also with the microorganisms infecting the ocular surface. Table 3 shows that besifloxacin administered in liposomes did not decrease the drug effect for both bacteria. In fact, for *P. aeruginosa*, the liposome with besifloxacin was able to exhibit lower values of MIC and MBC compared to the diluted commercial formulation, which contained a higher drug concentration. According DR% data (Table 1) LP PC and LP PC:SPM had losses in the process presenting recoveries 93 ± 0.5, and 68 ± 2.5%, respectively, meaning final besifloxacin concentrations ~ 7 and 32% lower than the controls formulation, which had 500 µg/mL of besifloxacin. The commercial formulation viscosity may have hampered drug release and permeation into the bacteria. Conversely, liposomal entrapment could maintain the same antibacterial effect as of the free drug in solution. *P. aeruginosa* is a gram-negative bacterium that produces higher amounts of extracellular polymeric substances^75^. Probably, these substances, which have excellent adhesion potential, could interact with the liposomes and enhance drug effect when compared to commercial formulation of the drug dispersed in a polymeric gel. Further studies are necessary to investigate these hypotheses. Also importantly, liposomes had the same efficiency as the free drug, demonstrating the controlled drug release does not affect the system's efficacy.

Besifloxacin is an antimicrobial drug known for having higher biologic effects against gram-positive and gram-negative bacteria than other fluoroquinolones^76^. In our case, MIC and MBC results suggest that *S. epidermidis* was more sensitive than *P. aeruginosa*. This observation is in agreement with Haas et al.^77^. The authors studied in vitro activity of besifloxacin against 2690 clinical isolates of ocular and respiratory origin from the United States. The results suggest lower MIC values for *S. epidermidis* than *P. aeruginosa*. Possibly, microbiological aspects, such as cell wall and resistance mechanisms, could explain this difference. Additional studies are needed to investigate this phenomenon.

## Conclusion

In this paper, we successfully incorporated besifloxacin into liposomes containing amines as positively charged additives and evaluated the influence of this charge on drug delivery under iontophoretic and passive delivery situations, in the latter, challenging the formulation residence with a simulated tear fluid. Iontophoresis seems to be a viable treatment strategy for a “burst drug delivery”, promptly providing higher drug bioavailability. However, positively charged additives do not provide additional enhancement. For the passive condition, besifloxacin incorporation into positively charged liposomes improved topical delivery with a formulation that is stable, safe, and conveniently easy to be instilled, which could improve topical ophthalmic treatments.

## Supplementary information

Supplementary Information.
