# Supplementary material for: Besifloxacin liposomes with positively charged additives for an improved topical ocular delivery
Source: Sci Rep. 2020 Nov 6;10:19285. doi: 10.1038/s41598-020-76381-y (PMC7648625; doi:10.1038/s41598-020-76381-y)
Supplement: Supplementary file 1 — Supplementary Information. [file 41598_2020_76381_MOESM1_ESM.pdf]

Besifloxacin liposomes with positively charged additives for an improved topical ocular delivery

Giselly A. dos Santos[1], Ricardo Ferreira-Nunes[1], Luciana F. Dalmolin[2], Ana Carolina dos Santos Ré[2], Jorge Luiz Vieira Anjos[3], Sebastião Antônio Mendanha[4], Carolina Patrícia Aires[2], Renata F.V. Lopez[2], Marcilio Cunha-Filho[1], Guilherme M. Gelfuso[1], Taís Gratieri[1] *.

SUPPLEMENTARY MATERIAL

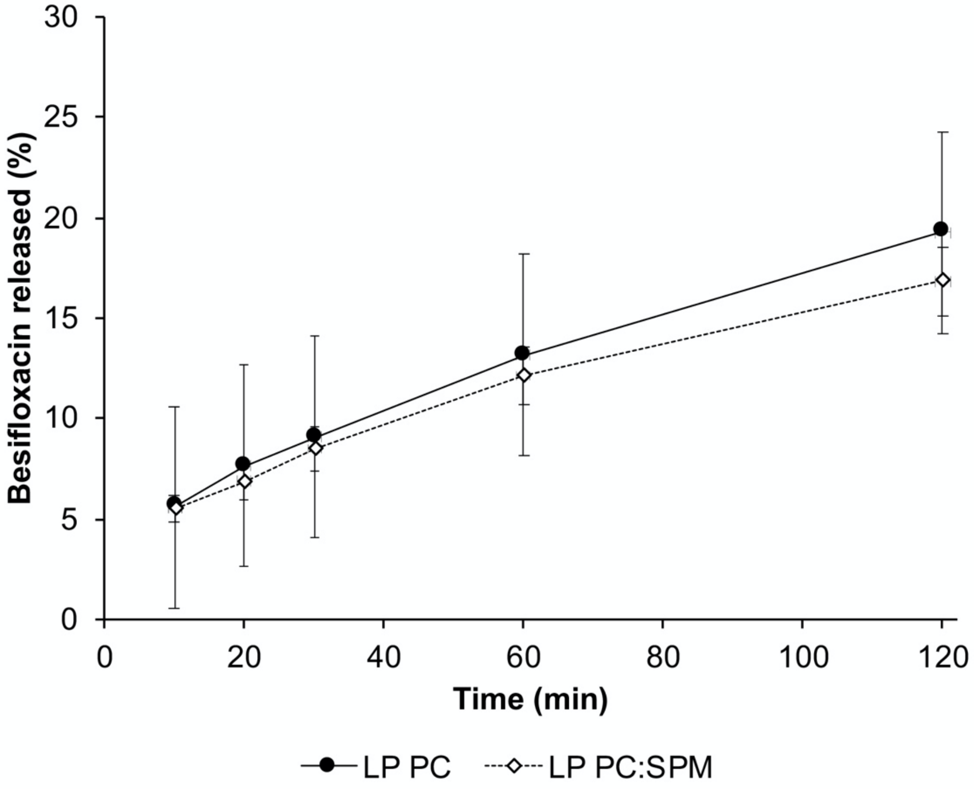

Supplementary Figure 1. Release profiles of besifloxacin from LP PC and LP PC:SPM for 2 h (n = 4). Data values were close to LOQ of the analytical method.

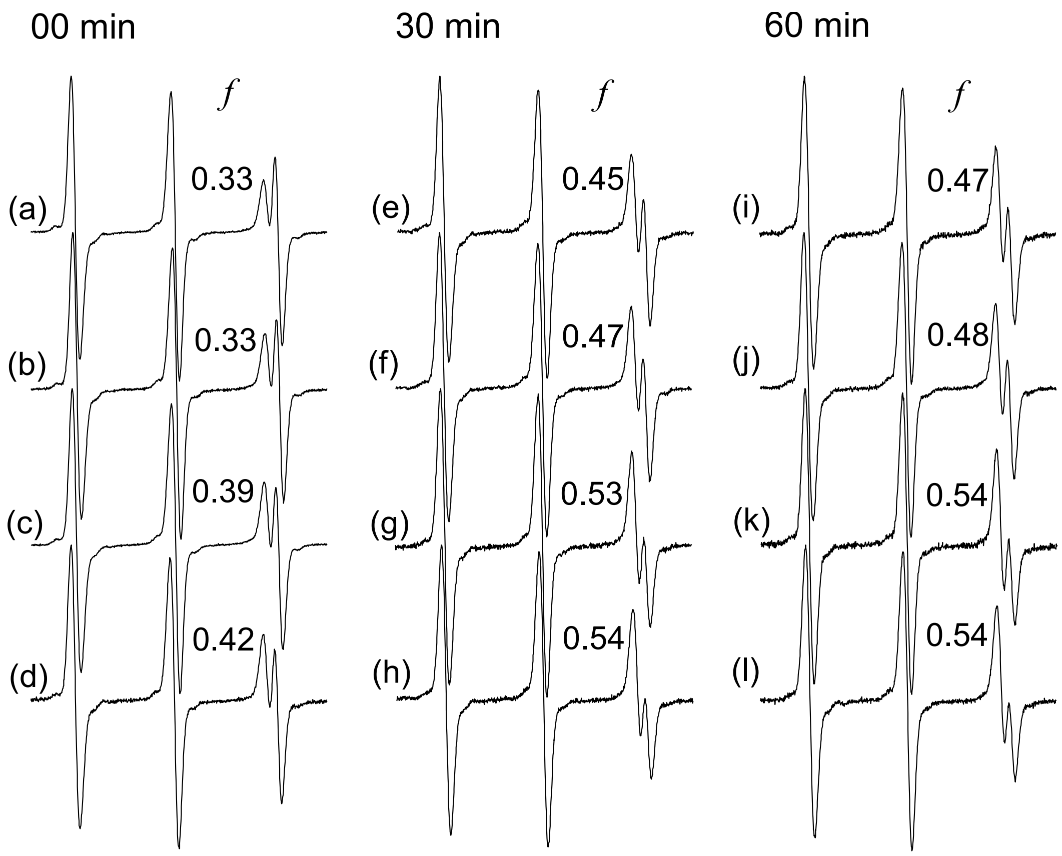

Supplementary Figure 2. Experimental EPR spectra of the TEMPO spin probe in liposomes of different composition at 32 °C and exposed to 2 mA of electric current intensity during 30 and 60 min. The values of the partitioning parameter *f* are indicated for each spectrum. The total magnetic field range used for all spectra was 50 G, and the intensities of the experimental spectra (on the y-axis) are normalized. Spectra (a), (e) and (i) refer to pure LP PC liposomes; spectra (b), (f) and (j) refer to LP PC-besifloxacin liposomes; spectra (c), (g) and (k) refer to LP PC:SPM liposomes; whereas spectra (d), (h) and (l) refer to LP PC:SPM-besifloxacin liposomes.

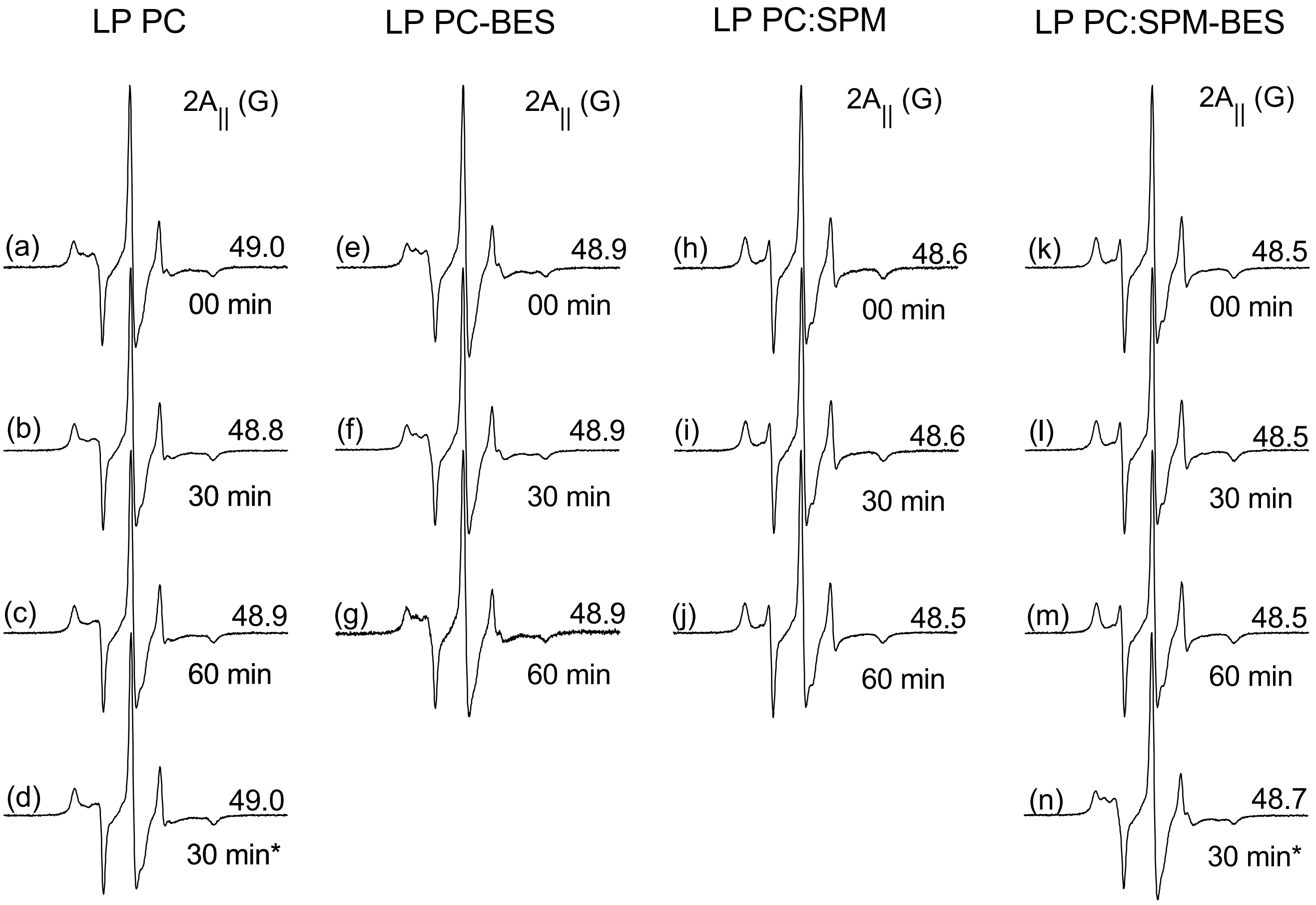

Supplementary Figure 3. EPR spectra of the 5-DSA spin label associated with different PC liposomes at 32 °C and exposed to 2 mA of electric current intensity during 30 and 60 min. The values of the maximum hyperfine splitting parameter $2A_{||}$ are indicated for each spectrum. The spectra of samples that were not exposed to electrical current are shown for comparison. The experimental error associated to the $2A_{||}$ values is 0.5 G. The intensities of the experimental spectra (on the y-axis) are normalized, and the total magnetic field range is 100 G. Spectra (d) and (n) refer to samples exposed to 4 mA of electric current during 30 min.
